# HDAC inhibitor AR-42 decreases CD44 expression and sensitizes myeloma cells to lenalidomide

**DOI:** 10.18632/oncotarget.5290

**Published:** 2015-09-25

**Authors:** Alessandro Canella, Hector Cordero Nieves, Douglas W. Sborov, Luciano Cascione, Hanna S. Radomska, Emily Smith, Andrew Stiff, Jessica Consiglio, Enrico Caserta, Lara Rizzotto, Nicola Zanesi, Volinia Stefano, Balveen Kaur, Xiaokui Mo, John C. Byrd, Yvonne A. Efebera, Craig C. Hofmeister, Flavia Pichiorri

**Affiliations:** ^1^ Department of Internal Medicine, Comprehensive Cancer Center, The Ohio State University, Columbus, OH, USA; ^2^ Department of Internal Medicine, Oncology/Hematology Fellowship, The Ohio State University, Columbus, OH, USA; ^3^ Lymphoma & Genomics Research Program, IOR Institute of Oncology Research, Bellinzona, Switzerland; ^4^ Department of Internal Medicine, Biomedical Sciences Graduate Program, Comprehensive Cancer Center, The Ohio State University, Columbus, OH, USA; ^5^ Department of Internal Medicine, Biosystems Analysis, LTTA, Department of Morphology, Surgery and Experimental Medicine, Università degli Studi, Ferrara, Italy; ^6^ Department of Neurological Surgery, Dardinger Laboratory for Neuro-oncology and Neurosciences, The Ohio State University Medical Center, Columbus, Ohio, USA; ^7^ Department of Biomedical Informatics, Center for Biostatistics, The Ohio State University, Columbus, OH, USA; ^8^ Department of Internal Medicine, Division of Hematology, The Ohio State University, Columbus, OH, USA; ^9^ Present Address: Sanford Burnham Prebys Medical Discovery Insitute, La Jolla, CA, USA

**Keywords:** myeloma, CD44, miR-9–5p, IGF2BP3, lenalidomide

## Abstract

Multiple myeloma (MM) is a hematological malignancy of plasma cells in the bone marrow. Despite multiple treatment options, MM is inevitably associated with drug resistance and poor outcomes. Histone deacetylase inhibitors (HDACi's) are promising novel chemotherapeutics undergoing evaluation in clinical trials for the potential treatment of patients with MM. Although in preclinical studies HDACi's have proven anti-myeloma activity, but in the clinic single-agent HDACi treatments have been limited due to low tolerability. Improved clinical outcomes were reported only when HDACi's were combined with other drugs. Here, we show that a novel pan-HDACi AR-42 downregulates CD44, a glycoprotein that has been associated with lenalidomide and dexamethasone resistance in myeloma both *in vitro* and *in vivo*. We also show that this CD44 downregulation is in part mediated by miR-9–5p, targeting insulin-like growth factor 2 mRNA binding protein 3 (IGF2BP3), which directly binds to CD44 mRNA and increases its stability. Importantly, we also demonstrate that AR-42 enhances anti-myeloma activity of lenalidomide in primary MM cells isolated from lenalidomide resistant patients and in *in vivo* MM mouse model. Thus, our findings shed light on potential novel combinatorial therapeutic approaches modulating CD44 expression, which may help overcome lenalidomide resistance in myeloma patients.

## INTRODUCTION

Multiple myeloma (MM) is a plasma cell (PC) neoplasm that accounts for more than 20,000 new cases every year in the United States [1–3]. Development of novel therapeutic options, such as proteasome inhibitors (PI) and immunomodulatory agents (IMiDs), has improved treatment outcomes. Patients eligible for transplantation show 5 year survival in more than 70% of the cases, which is reduced to ~50% in the transplant ineligible subjects [4, 5]. However, the overall survival of patients carrying high-risk MM cytogenetic abnormalities is still very poor and they inevitably relapse [3]. Alternative novel treatment strategies are therefore urgently needed [6–9]. Epigenetic modifications such as DNA methylation and histone acetylation, as well as microRNA deregulation play important roles in the pathogenesis and treatment responses of MM [10–13].

Histone acetyltransferases and histone deacetylases (HDACs) affect a broad-array of genes involved in cell cycle, apoptosis, and protein folding [14]. The first FDA-approved deacetylase inhibitor (HDACi), suberoylanilide hydroxamic acid (SAHA, Vorinostat), was shown to be effective *in vitro* and to have clinical efficacy in T-cell lymphomas [15]. However, in MM it showed only minimal activity as a single agent [16]. For most HDACi's the mechanism of action in MM is unknown, but at biologically achievable concentrations, it has been theorized that HDACi's can sensitize MM cells to other drugs by interfering with cell adhesion mediated drug resistance (CAM-DR) [17–19]. Indeed, in two phase 1 trials some patients were able to be salvaged by a combination of HDACi's (SAHA, or panobinostat) with proteasome inhibitor, bortezomib [20, 21]. Also phase 1/2 studies of combination of SAHA, or panobinostat with lenalidomide have demonstrated tolerability and activity in lenalidomide-refractory patients [22, 23]. Recently, a novel orally bioavailable class I/II, phenylbutyrate-based HDAC inhibitor, AR-42 (ARNO Therapeutics, Parsippany, NJ) has been developed and shown to have a greater anti-proliferative effects, as compared to SAHA, both *in vitro* and *in vivo* [24]. One of the biological effects of AR-42 is that it is able to inhibit activation of STAT3 even in the presence of interleukin (IL)-6 activation signal and thus, induce apoptosis of MM cells [25].

Dexamethasone and lenalidomide resistance in MM has been attributed to upregulation of CD44 [26], which is a cell surface glycoprotein playing roles in cell adhesion, migration and cell-cell interactions [27]. It functions as a receptor for hyaluronic acid, which itself is considered a tumor marker in cancer [28, 29]. Moreover, CD44 forms a complex with STAT3 and p300 (acetyltransferase) causing STAT3 activation in a cytokine- and growth factor-independent manner [30]. Thus, pharmacological targeting of CD44 may affect different pathways in MM malignancies and be beneficial for dexamethasone- and lenalidomide-resistant patients.

Here, we demonstrate that AR-42 down-regulates CD44 protein and mRNA levels *in vitro* and *in vivo*. We found that the molecular mechanism, by which AR-42 is able to decrease CD44 expression is through the up-regulation of miR-*9-5p*, which directly targets and down-regulates the RNA binding protein IGF2BP3, known to physically bind to CD44 mRNA and increase its stability [31]. Furthermore, we show that in a mouse model, AR-42 it is able to increase the sensitivity of MM cells to lenalidomide and the combination of both drugs has a synergistic effect.

## RESULTS

### AR-42 down-modulates CD44 in myeloma cells

**Figure 1:**
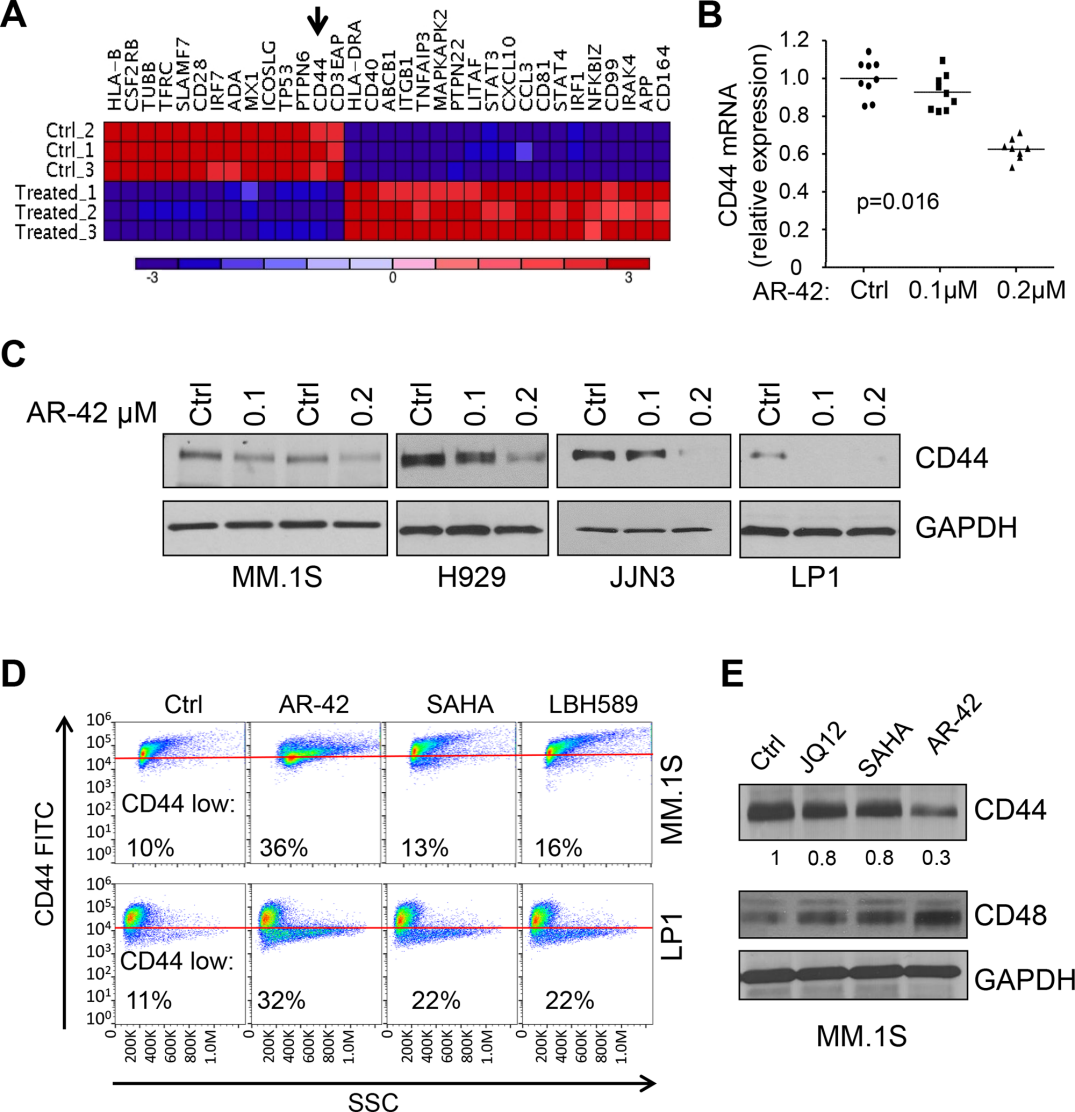
AR-42 treatment induces CD44 downregulation in multiple myeloma cell lines **A.** Dendrogram of the unsupervised hierarchical clustering analysis of nCounter^®^ GX Human Immunology assays in MM.1S cells treated with 0.1 μM AR-42 for 24 hrs. **B.** CD44 mRNA expression measured by qRT-PCR in RNA from MM.1S cells treated for 24 hrs with 0.1, or 0.2 μM AR-42. GAPDH was used for normalization. **C.** CD44 protein expression in MM.1S, H929, JJN3 and LP1 cells treated with AR42 at 0.1 and 0.2 μM, or vehicle control (Ctrl) for 24 h. GAPDH was used as a loading control. **D.** Flow cytometric analyses of the CD44 expression in MM.1S and LP1 cells upon 24 h treatment with AR42 (0.2 μM), SAHA (1 μM) and LBH589 (LBH; 0.01 μM). Events expressing low level of CD44 are shown as % of the total events. All experiments were performed in triplicate. **E.** MM.1S cells were treated with different HDACi's (0.5 μM JQ12, 1 μM SAHA, or 0.2 μM AR-42), or vehicle control (Ctrl) for 48 hrs and analyzed by western blot for the levels of CD44 protein. The same blot was also stained with anti-CD48 antibody and the results were normalized to GAPDH.

Growth inhibitory and pro-apoptotic properties of pan-HDACi, AR-42, have been reported previously [25, 32, 33] in numerous malignancies, including MM. Because of the potent immunomodulatory effects observed with classical pan-HDACi's [34], we investigated whether immunology-related gene networks were altered upon AR-42 treatment in MM cells. We used nCounter technology to analyze the effects of AR-42 on the expression of 511 human genes in MM.1S cells. We chose a 24-hr treatment with 0.1 μM AR-42, because we found that this treatment leads to a detectable hyperacetylation of histone 3 and 4 (Supplementary Figure S1A), but without significant increase of apoptosis, as measured by Annexin V-PI staining in all MM cell lines tested (MM.1S, U266, RPMI-8226, MM.1R) (Supplementary Figure S1B and data not shown). Unsupervised hierarchical clustering analysis identified two distinct branches corresponding to AR-42 treated and untreated cells (Figure 1A), and showed that expression of numerous immunology-related genes was strongly altered (Supplementary Table S1). Among the most significantly downregulated genes (*p* < 0.001) were several cell membrane associated proteins, including CD44 (Supplementary Table S1).

We focused on CD44 expression, because in MM cells its expression correlates with cell adhesion mediated drug resistance (CAM-DR) [17–19] and it has been shown to mediate resistance to dexamethasone [35] and lenalidomide [26]. Using qRT-PCR validation, we found that CD44 mRNA (Figure 1B) and protein levels (Figure 1C, Supplementary Figure S1C) were consistently downregulated by 24-hr treatment with AR-42 in a dose-dependent fashion, as compared to the vehicle control (DMSO; Ctrl). Reduction of CD44 mRNA and protein persisted for 48 hrs after treatment (Supplementary Figure S1C, S1D and data not shown). The down-regulation of CD44 cell surface expression was also observed by flow cytometry in all MM cell lines tested expressing detectable CD44 levels (Figure 1D, Supplementary Figure S1E, S1F and data not shown). Of note, at 48 hrs of AR-42 treatment we observed a consistent up-regulation of CD48 at protein and mRNA levels (Figure 1E and data not shown), supporting the idea that AR-42 mediated CD44 down-regulation is not simply associated with a global down-regulation of the surface molecules of MM cells. We also compared the effect of AR-42 with other HDACi's in clinical use and we found that cells treated with AR-42 showed greater CD44 downregulation, when compared with SAHA, LBH589 and HDAC1/2 inhibitor (JQ12) and used at comparable IC_50_ concentrations (0.2 μM AR-42, 1.0 μM SAHA, 0.01 μM LBH, and 0.5 μM JQ12) (Figure 1D–1E, Supplementary Figure S1G).

### AR-42 decreases CD44 levels *in vivo*

**Figure 2:**
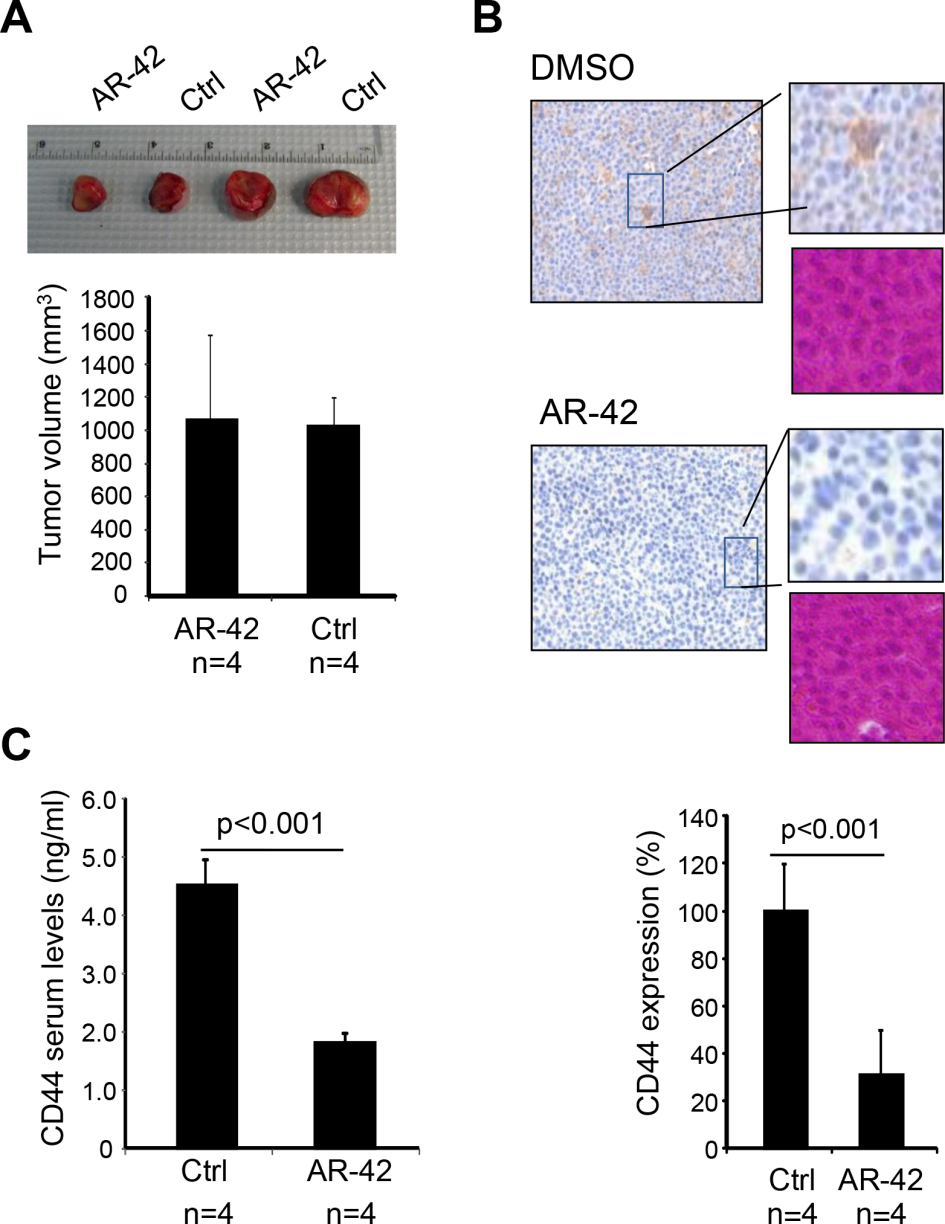
AR-42 decreases CD44 expression *in vivo* **A.** MM.1S cells were injected subcutaneously into nude mice and 3 weeks later mice were divided into 2 groups (4 mice per group), such that overall tumor sizes were comparable between the groups. Following 2 doses of AR-42 treatment (25 mg/kg), or vehicle control (Ctrl) the tumors were excised and measured. **B.** Tumors were tested for the expression of CD44 by IHC. Positive staining of CD44 is seen as brown color. Hematoxylin and eosin was used to counter-stain sections. **C.** Soluble CD44 serum levels from xenografted mice treated with 2 doses of AR-42 (or Ctrl) were quantified by ELISA.

To investigate if AR-42 treatment could affect CD44 expression *in vivo*, we created a xenograft MM mouse model by injecting 1 × 10^7^ viable cells of MM.1S cell line subcutaneously into the right flank of 12 NOD-SCID mice. Three weeks later, a group of 8 mice containing comparable tumor size (250 ± 60 mm^3^) were selected and randomly divided into 2 groups. One group of mice (*n* = 4) received intra-peritoneal injections of 25 mg/kg AR-42, while the second group (*n* = 4) was injected with vehicle control (8% DMSO in PBS; Ctrl). Injections were administered once a day (on Monday and Wednesday). Because the anti-tumor activity of AR-42 has been previously reported in preclinical mouse studies [33], in order to avoid tumor size reduction mice were sacrificed 2 days after the second injection. Indeed, at this time point the tumors were still comparable between the mouse groups (Figure 2A). Tumors were excised and used for CD44 immunohistochemical (IHC) studies, while the serum was collected for ELISA assays. IHC analysis of tumor sections revealed that the AR-42-treated mice displayed significant lower CD44 staining compared with the control group (Figure 2B). ELISA assays also showed decreased levels of soluble CD44 in the serum of the mice treated with AR-42 (Figure 2C). In conclusion, our data demonstrate that AR-42 is able to down-regulate CD44 directly *in vivo*.

### AR-42 modulates expression of microRNAs in MM cells

**Figure 3:**
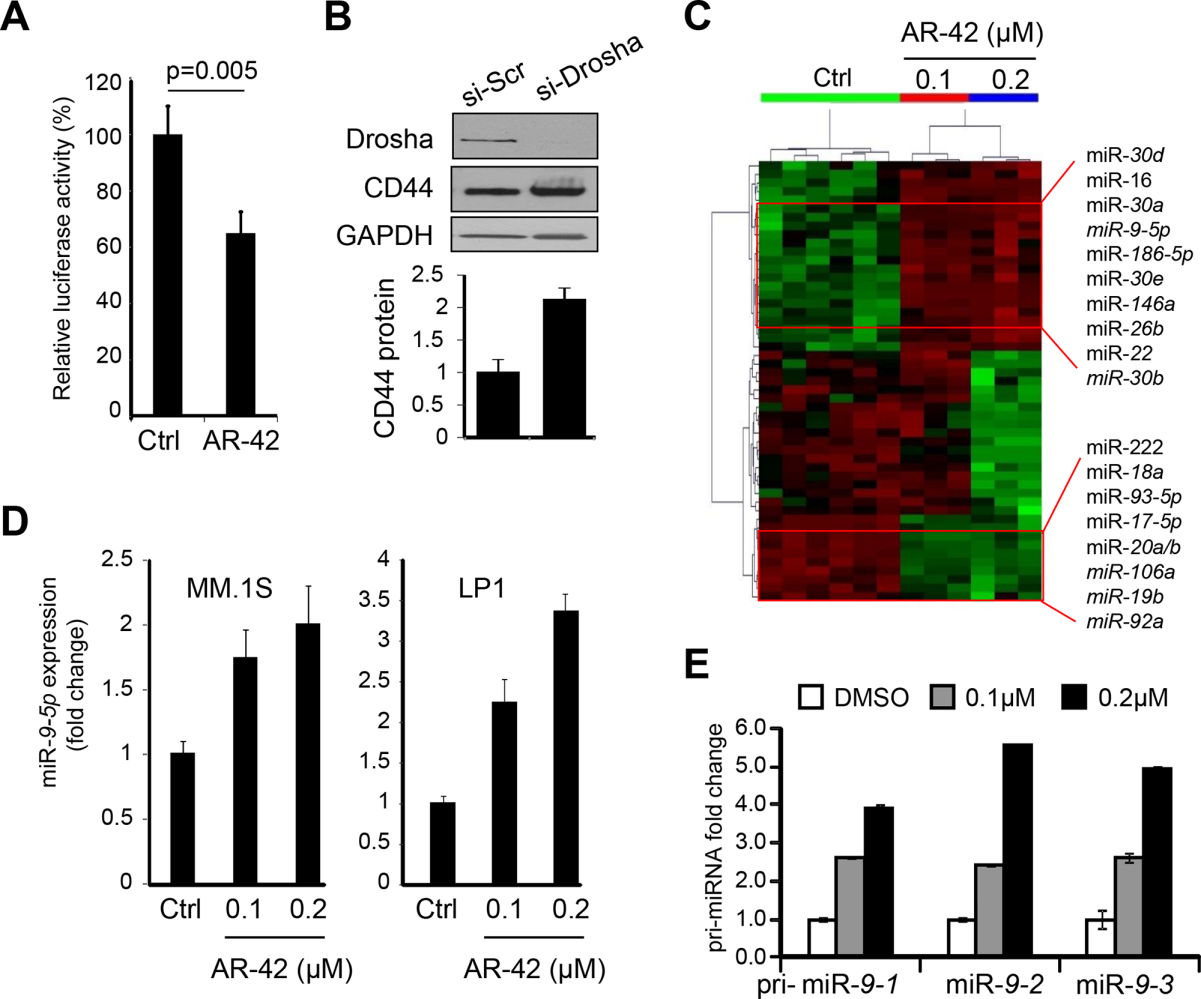
AR-42 upregulates expression of miR-9-5p **A.** Luciferase assay in MM.1S cells transiently transfected with pGL3-CD44 3′UTR construct and treated for 24 hrs with 0.2 μM AR-42, or vehicle control (Ctrl) showing inhibitory response to AR-42 *via* 3′UTR element. Each measurement was done in triplicate. **B.** MM.1S cells were treated with RNA silencing for Drosha (si-Drosha) or unrelated sequence (si-Scr). Forty eight hours later, cells were lysed and analyzed by western blot using anti-Drosha and anti-CD44 antibodies. GAPDH was used for normalization. Signals were quantified using ImageJ and plotted below. **C.** Dendrogram of the unsupervised hierarchical clustering analysis of global miRNA expression in MM.1S cells treated with designated concentrations of AR-42, or vehicle control (Ctrl), using NanoString technology. Selected most up-regulated (upper) and down-regulated (lower) miRNAs are indicated. **D.** miR-*9-5p* expression in MM.1S (left) and LP1 (right) cells treated with AR-42 at 0.1 and 0.2 μM, or vehicle control (Ctrl) was determined by qRT-PCR. Results are expressed as fold change compared to the DMSO (Ctrl). **E.** The effect of 24-hr treatment of MM.1S cells with AR-42 (at indicated concentrations) on expression of pri-miR-*9-1*, pri-miR-*9-2* and pri-miR-*9-3* was determined by qRT-PCR.

To address the molecular mechanism(s) responsible for downregulation of CD44 gene, we considered the potential role of one or more *cis* regulatory regions. However, to our surprise 24 hr treatment with 0.2 μM of AR-42 did not lower the activity of CD44 promoter region in MM cells (MM.1S, U266 and 293T ) (Supplementary Figure S2). Therefore we turned our attention to the 3′UTR of CD44 and asked if inhibition of CD44 expression by AR-42 might be mediated by microRNA(s) potentially upregulated by AR-42 treatment. As the first step, we subcloned CD44 3′UTR element downstream from the SV40 promoter-driven luciferase gene and transiently transfected the resulting reporter plasmid into MM.1S cells. Incubation with 0.2 μM AR-42 for 24 hrs resulted in 35% decrease in luciferase activity, as compared to untreated cells (Figure 3A).

Since RNA ribonuclease, Drosha is critical during the initial steps of microRNA processing [36], we tested the effect of Drosha knock-down on CD44 expression using specific siRNA. Figure 3B demonstrates that inhibition of Drosha expression in MM.1S cells resulted in 2-fold increase of CD44 protein levels. Thus, these results support the idea that the down-modulation of CD44 expression by AR-42 is mediated by CD44 3′UTR and it may involve upregulation of microRNA(s) targeting CD44 3′UTR.

To determine if miRNAs are regulated by AR-42 in MM cells at sub-lethal concentrations, we performed a full-spectrum analysis of miRNA levels using NanoString technology [37] with an expanded set of probes capable of assaying the expression of more than 800 human miRNAs. We performed an array analysis of global miRNA expression on RNA from MM.1S cells grown in the presence or absence of AR-42 at different concentration (0.1, or 0.2 μM) for 24 hours. Unsupervised hierarchical clustering analysis produced a dendrogram, in which samples are segregated according to class of treatments (Figure 3C). We found that 51 miRNAs were differentially expressed between the two groups, of which 29 were significantly down-regulated in cells treated with AR-42 and the other 22 were up-regulated (Supplementary Table S2). Since we were interested in defining the mechanism of decreasing of CD44 expression by AR-42 via microRNA(s), we focused on miR's upregulated by the treatment. Stem loop real time PCR (qRT-PCR) was used to validate the most-upregulated miRNAs in several cell lines (MM.1S, LP1, H929, and JJN3), which revealed that miR-*9-5p* had the strongest response to the treatment and its expression levels increased at 24 hrs in a dose dependent manner when compared to the control treatment (Figure 3D).

Because human miR-*9-5p* is encoded by three distinct genomic loci, specifically primary (pri)-miR-*9-1* on chromosome 1 (q22), pri-miR-*9-2* on chromosome 5 (q14.3), and pri-miR-*9-3* on chromosome 15 (q26.1), we investigated which locus was responsible for miR-*9* up-regulation in response to AR-42. Quantitative RT-PCR showed dose dependent changes in all primary transcripts of miR-*9-5p* in AR-42-treated MM.1S cells, as compared to controls, supporting the idea that all these chromosomal regions contribute to miR-*9-5p* up-regulation upon AR-42 in MM cells (Figure 3E).

### The CD44 mRNA binding protein IG2FBP3 is the direct target of miR-*9-5p*

**Figure 4:**
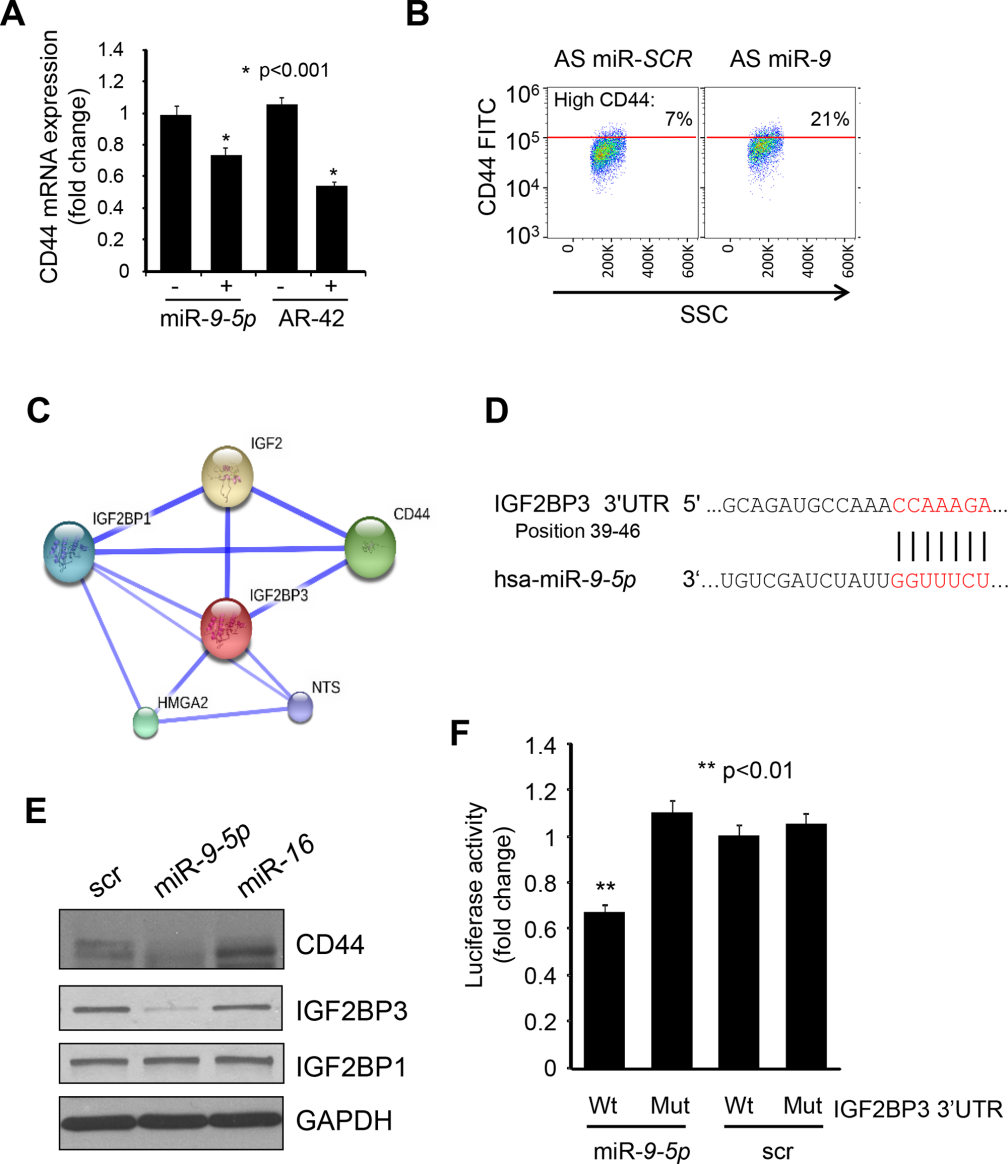
IGF2BP3 is the direct target of miR-9-5p **A.** MM.1S cells were transfected with miR-*9-5p*, or negative control miR precursor (Ctrl) and analyzed for the expression of CD44 mRNA by qRT-PCR. For comparison, U266 cells treated with 0.2 μM AR-42 (+), or vehicle control (−) were also included in the analysis. **B.** Bivariate dot plot of the CD44 expression in MM.1S cells transfected with AS miR-*SCR*, or AS miR-*9* and determined by flow cytometry. The percentages indicate the amount of events expressing the highest level of CD44. **C.** Diagram generated based on STRING database showing functional interaction networks between IGF2BP1, IGF2BP3, and CD44 **D.** IGF2BP3 3′UTR contains seed sequence for miR-*9-5p* (indicated in red). **E.** L363 cells were transfected with miR-*9-5p*, miR-*16*, or scramble control and 48 hrs later 80 μg of protein extract was analyzed by western blot for the levels of IGF2BP3 and IGF2BP1. GAPDH served to normalize the data. **F.** The luciferase reporter genes containing IGF2BP3 3′UTR, either wild type (Wt), or mutant at the predicted miR-*9-5p* binding site (Mut), were cotransfected with miR-*9-5p* precursor, or negative control miR (scr) into MM.1S cells. Luciferase assay was performed 24 hrs later and the results are expressed as fold change of Wt construct cotransfected with scr miR.

To determine if CD44 is a direct target of miR-*9-5p*, we performed a bioinformatic search (Target Scan [38], Pictar [39], and miRDB) for predicted miR-*9-*5p binding site(s) in CD44 3′UTR, but we didn't find any (data not shown). Moreover, none of the microRNAs identified in our NanoString assay to be upregulated by AR-42, were predicted to bind to CD44 3′UTR (data not shown). Therefore, we considered that miR-*9-5p* may regulate CD44 expression in an indirect fashion. To test this hypothesis, we transiently transfected MM.1S cells with miR-*9-5p* precursor, or scramble control, and measured CD44 mRNA levels by qRT-PCR 48 hrs later. Figure 4A shows that in the miR-*9-5p* transfected cells (+) CD44 expression is about 30% lower when compared to scramble control transfected cells (−). In the reciprocal experiment, MM.1S cells were transiently transfected with antago-miR-*9* (AS miR-*9*), or scramble control (AS miR-SCR) and CD44 surface expression was measured. As demonstrated in Figure 4B, inhibition of the endogenous miR-*9-5p* increased more than two times the population with high CD44 expression when compared to cells transfected with scramble sequence (SCR). Taken together, these results lend support to a critical role of miR-*9-5p* in regulating CD44 expression in MM cells.

One possible mechanism could involve the regulation of CD44 mRNA stability. Therefore, we focused our attention on two related RNA binding proteins, IGF2BP1 and IGF2BP3, which bind and control CD44 mRNA stability [40] in several cellular system and their expression is tightly related to CD44 levels in several forms of cancer [41]. STRING data analysis (http://string-db.org) shows strong functional interaction between IGF2BP1, IGF2BP3, and CD44 (Figure 4C). Using Targetscan [38], Pictar [39], and RNA22 [42] searches we identified a highly conserved consensus sequence for miR-*9-5p* in the 3′UTR of IGF2BP3, and a lower probability site in the 3′UTR of IGF2BP1 (Figure 4D and data not shown). To test if IGF2BP1 and IGF2BP3 are *bona fide* targets of miR-*9-5p*, we transfected MM cells with miR-*9-5p* precursor, or scramble control and measured IGF2BP1 and IGF2BP3 protein expression by western blot. In agreement with the prediction, ectopic expression of miR-*9-5p* led to a strong decrease of IGF2BP3 protein paralleled by downregulation of CD44 protein, while the expression of IGF2BP1 was not affected (Figure 4E). Ectopic expression of another microRNA identified in our NanoString experiment (miR-*16*) did not influence the protein levels of IGF2BP3, CD44, or IGF2BP1 (Figure 4E), thus demonstrating the specific effect of miR-*9-5p*. To examine if miR-*9-5p* targets IGF2BP3 directly, we cloned the portion of the IGF2BP3 3′UTR containing either the wild type (Wt) or mutated (Mut) miR-*9-5p* site into a pGL3-control luciferase vector. Luciferase activity significantly decreased when the Wt reporter construct was cotransfected into MM.1S cells with miR-*9-5p*, as compared to scramble control (scr) (Figure 4F). This effect was not observed when IGF2BP3 3′UTR with a specific deletion of 2 nucleotides (Mut) in miR-*9-5p* consensus sequence was tested (Figure 4F). In summary, these data indicate that CD44 expression can be modulated by changes in miR-*9-5p* levels, although indirectly. Furthermore, we also discovered that miR-*9-5p* directly targets IGF2BP3 (but not IGF2BP1), a stabilizer of CD44 mRNA [40].

### AR-42 treatment sensitizes MM cells to lenalidomide

**Figure 5:**
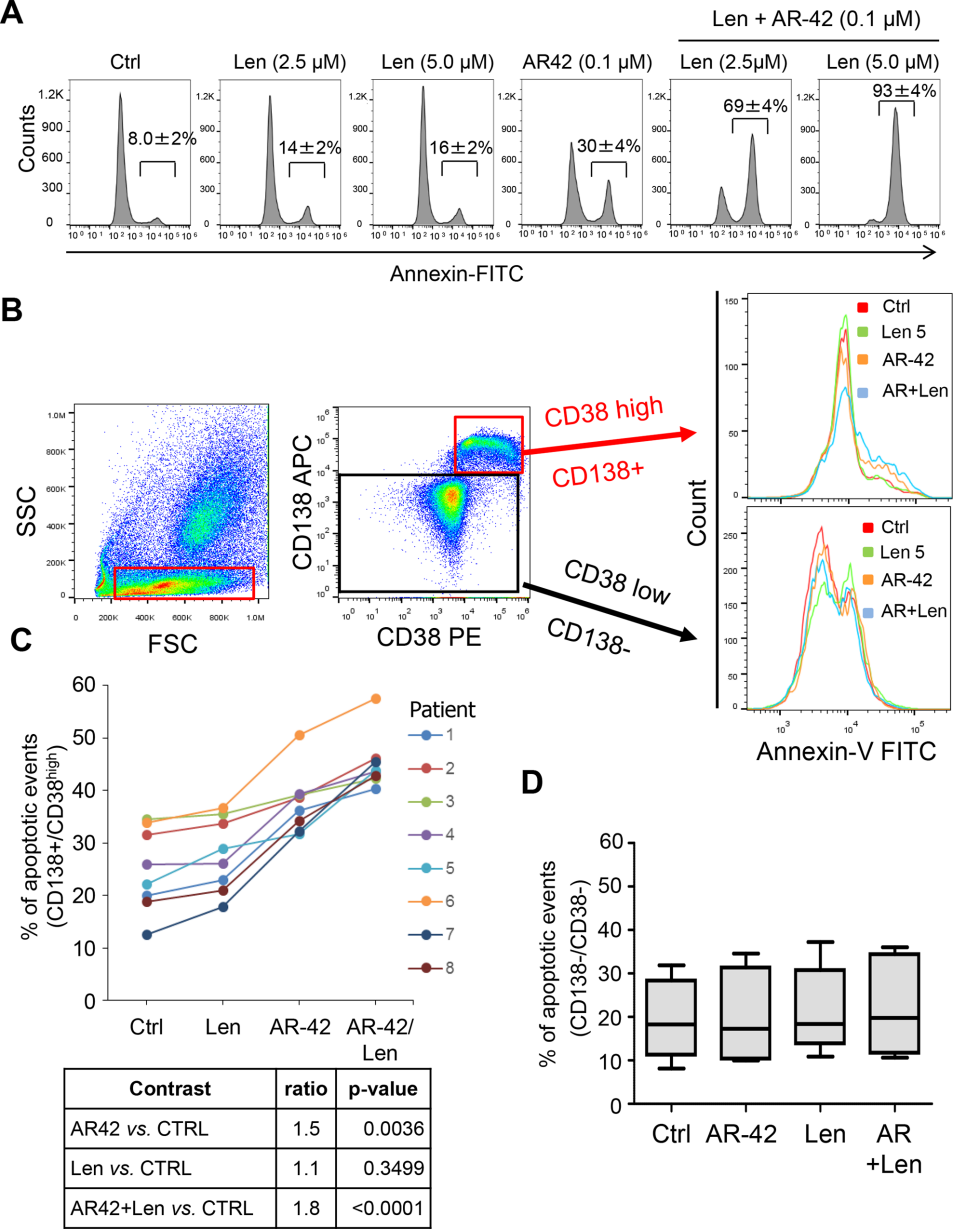
The effect of combined AR-42 and lenalidomide treatment in MM cell lines and primary MM patient bone marrow-derived cells **A.** Annexin V expression by flow cytometry in MM.1S cells treated twice (every 24 hours), with Len (2.5, or 5.0 μM), AR-42 (0.1 μM), or combination Len+AR-42, as indicated. Values represent the average percentages of positive events ± SD from three independent experiments. **B.** Strategy of analysis of apoptosis by flow cytometry in bone marrow samples. Bone marrow cells from 5 Len-refractory and 3 newly diagnosed MM patients were stained with anti-CD138 and anti-CD38 antibodies and sorted into 2 populations: CD38^high^/CD138+ (MM plasma cells) and CD38^low^/CD138-. Each population was further divided and treated with vehicle control (Ctrl), 5 μM Len, 0.25 μM AR-42, a combination of both drugs for 48 hrs, followed by flow cytometric Annexin V apoptosis assay. **C.** Annexin-V induction in CD38^high^/CD138+ MM cells treated as described in (B) Data are expressed as % of Annexin V positive events. **D.** Flow cytometric evaluation of apoptosis in CD38^low^/CD138- BM population from the same MM patients, as in (C) Data are expressed as % of Annexin V positive events.

Recently published data have shown that CD44 up-regulation in MM cells is associated with resistance to lenalidomide [26]. Given the fact that AR-42 inhibits expression of CD44 (Figures 1 and 2), we hypothesized that AR-42 can sensitize MM cells to lenalidomide treatments. Hence, we examined whether AR-42 treatment leads to increased apoptosis in MM.1S cells upon exposure to lenalidomide (Len). Indeed, as revealed by Annexin V staining of MM.1S and MM.1R cells (Figure 5A and data not shown), the addition of AR-42 (0.1 μM) to Len (2.5 μM) resulted in a 4.9-fold increase in apoptosis at 48 hrs, relative to Len alone, while the combination of 0.1 μM AR-42 and 5.0 μM Len resulted in a 5.8-fold increase (Figure 5A). Next, we treated MM.1S cells with 0.1 and 0.2 μM AR-42 in combination with different concentrations of Len (1–10 μM) and measured their effects cell proliferation assay (MTT). To calculate combination indices (CI) we utilized the Chou-Talalay method [43]. We found that the combination of AR-42 with Len showed strong synergism (CI < 1) in killing of MM.1S cells (Supplementary Table S3).

Since MM cell survival is strongly dependent on microenvironment [44–46], we decided to assess whether AR-42 in combination with Len can increase MM cell killing in the context of the bone marrow (BM) milieu. Total BM samples obtained from 5 Len-refractory MM (patients 1, 2, 3, 4, and 7) and 3 newly diagnosed MM patients (patients 5, 6, and 8) were treated with AR-42 (0.2 μM) and Len (5 μM) as single agents, and in combination. Following 48 hrs of treatment, multiparametric flow cytometry (diagrammed in Figure 5B) showed a substantial increase of Annexin V staining, specifically in the CD138+/CD38^high^ MM cells [47, 48] treated with AR-42 in combination with Len (*p* < 0.0001) (Figure 5C). In contrast, the CD138^neg^/CD38^low^ BM cellular fraction did not demonstrate significant evidence of apoptosis following the combination treatment (Figure 5D). Also peripheral blood mononuclear cells (PBMCs) in the same experimental conditions did not show induction of apoptosis (Supplementary Figure S3). The BM cells from the same patients were also used to assess CD44 expression at 24 hrs after AR-42 treatment. Downregulation of CD44 was observed in the whole BM of all 5 Len-refractory and 2 of the 3 newly diagnosed MM patients (patients 5 and 8) (Supplementary Figure S4).

### AR-42 treatment sensitizes MM cells to lenalidomide *in vivo*

**Figure 6:**
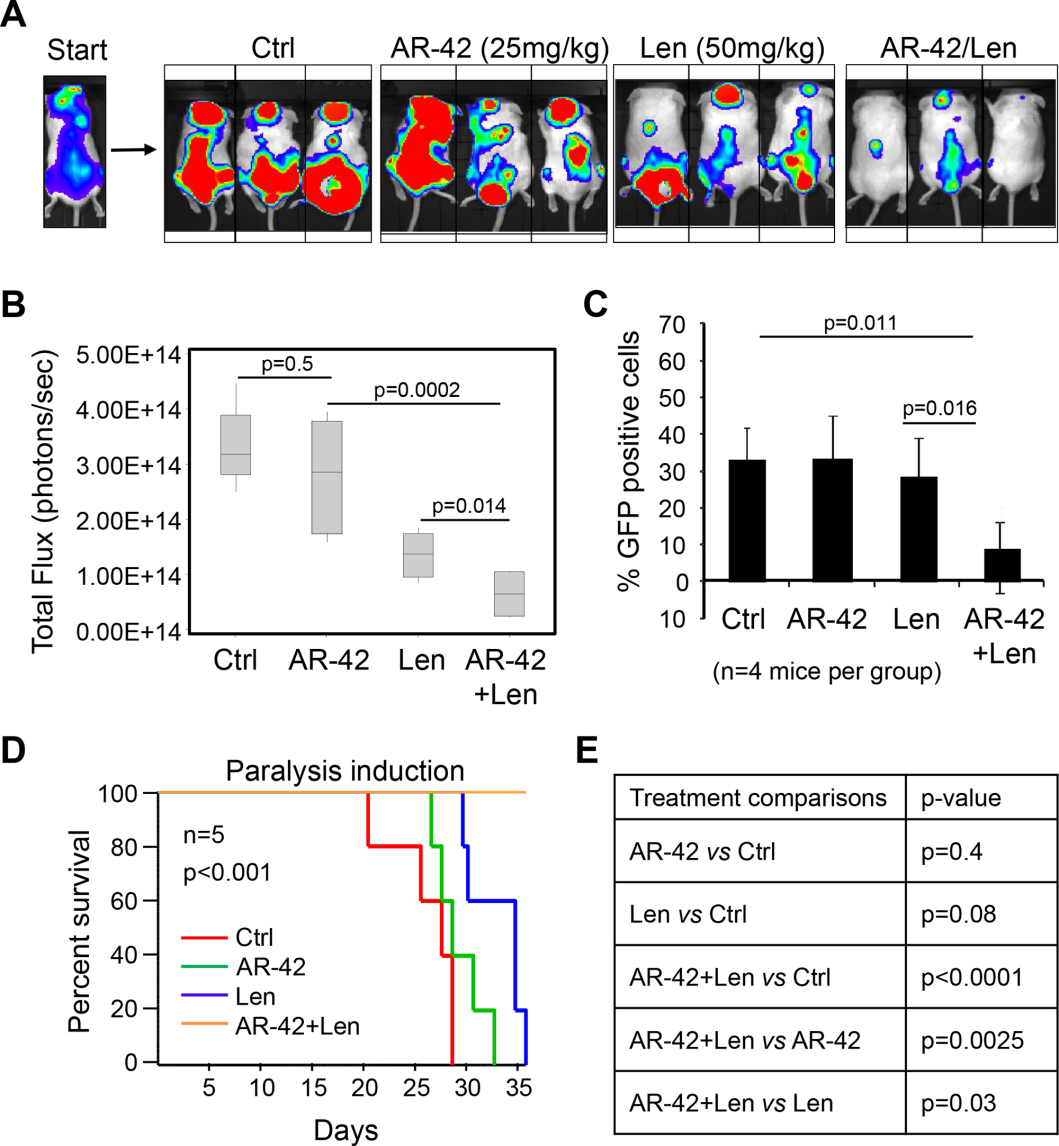
Synergistic effect of AR-42 and lenalidomide combination therapy *in vivo* **A.** NOD-SCID mice engrafted with 5 × 10^6^ MM.1S GFP+/Luc+ cells and treated for 3 weeks with vehicle control (Ctrl), AR-42, lenalidomide (Len), or AR-42/Len combination. Representative luminescence images are shown. **B.** Tumor progression was evaluated by bioluminescence imaging, which showed that the tumor growth was markedly suppressed in AR-42+Len treated mice, compared to control group (Ctrl), or single agent treated mice. **C.** Analysis of murine BM tumor progression by CD138-GFP+ quantification by flow cytometry. Data represent the mean ± SD of each group of treatment. **D.** Kaplan-Meier survival curves for mice in each group of treatments. Comparison between the different groups of treatments was made *p*-values associated with survival are shown in **E.**

To investigate the effect of AR-42 and Len *in vivo*, NOD-SCID mice (*n* = 40) were intravenously injected with 5 × 10^6^ GFP+/Luc+ MM.1S cells [49]. After three weeks, mice with similar tumor burden were selected and divided into 4 treatment groups (5 mice per group): AR-42 alone, Len alone, AR-42/Len, and vehicle control (8% DMSO in PBS; VE). To minimize toxicity and investigate a clinically relevant treatment regimen, mice were treated with Len (50 mg/kg) or VE by intraperitoneal injections daily, and AR-42 (25 mg/kg) or VE 3 times per week for 3 weeks. Following treatments, tumors were markedly suppressed in all AR-42/Len treated mice compared to control and single agent [AR-42 *vs*. Ctrl (*p* = 0.5); Len *vs*. Ctrl (*p* = 0.014); AR-42/Len *vs*. Len (*p* = 0.0145); AR-42/Len *vs*. AR-42 (*p* = 0.0002)] (Figure 6A–6B). The extent of BM engraftment was determined by flow cytometry using a human anti-CD138 antibody, and it was evident that AR-42/Len treated mice showed significantly less BM engraftment compared to the other treatment groups [AR-42 *vs*. Ctrl (*p* = 0.8); Len *vs*. Ctrl (*p* = 0.8); AR-42/Len *vs*. Len (*p* = 0.016); AR-42/Len *vs*. AR-42 (*p* = 0.01)] (Figure 6C). All mice treated with the AR-42/Len combination displayed a longer survival when compared to the mice treated with single agent. In fact, they all appeared healthy and remained alive past the point, at which the last mice in all other treatment groups were removed (Figure 6D–6E). Thus our data indicate that AR-42 in combination with lenalidomide can be a valid therapeutic strategy to increase lenalidomide sensitivity of MM cells in the BM niche.

## DISCUSSION

Adhesion molecules play a key role in mediating the interaction between MM cells and the extracellular environment in bone marrow, including stromal cells and the extracellular matrix [50, 51]. They also strongly contribute to MM resistance to therapeutic intervention. Many primary CD138+ MM cells have detectable surface expression of CD44, CD49d, and CD54 (ICAM-1) [50, 51], and which of those adhesion molecules is the most important remains controversial. CD44 is the major cell surface receptor for hyaluronan (HA) [52] and this interaction is important for diverse biological functions, such as cell proliferation, adhesion, migration, and angiogenesis [52, 53]. High levels of CD44 are associated with drug resistance and poor prognoses in various malignancies, mainly due to its physical association with P-glycoprotein (Pgp), a product of the multidrug resistance gene 1 (MDR1) [54–56].

CD44 is a downstream target of Wnt signaling [57] and is considered one of the most important markers of cancer stem cells (CSCs) [58]. Since HA is a major component of the BM niche in MM patients, HA-CD44 interaction could be critical in regulating myeloma CSC functions leading to increased myeloma CSC self-renewal and chemoresistance, as shown in other forms of cancer [59, 60]. Hence, we can speculate that the downregulation of CD44 expression upon pan-HDACi treatment has the potential to sensitize to therapy myeloma CSC in the bone marrow niche, a hypothesis that needs further evaluation.

The importance of CD44 expression in treatment-resistance of MM has been highlighted by the demonstration that blocking CD44/HA interaction resulted in protecting dexamethasone-induced apoptosis [35]. Furthermore, in a recent *in vitro* study, Bjorklund et al. [26] showed the critical role played by CD44 in resistance to lenalidomide. Thus, therapeutic interventions involving regulation of CD44 provide an attractive strategy to overcome the drug resistance.

Interestingly, our NanoString profiling of the genes affected by a novel HDACi, AR-42, identified CD44 as one of the genes downregulated by the drug. This is particularly important, since AR-42 is currently being tested in phase I clinical trials for hematologic malignancies and solid tumors [61]. Here we found that AR-42 treatments down-modulate CD44 expression and sensitize MM cells to lenalidomide. Although other HDACi's can also increase the sensitivity to lenalidomide affecting MM cell line proliferation properties, as recently published [62], we show that clinically achievable concentrations of AR-42 display a greater ability to downregulate the drug resistance marker, CD44 when compared to other pan-HDACi's, not only in cell lines but also in primary cells isolated from lenalidomide resistant patients. Hence, we hypothesize that AR-42 may be more suitable as an IMiD companion drug to overcome mechanisms of resistance in relapsed patients. To understand the molecular mechanism(s) responsible for AR-42 induced inhibition of CD44 expression, we investigated the involvement of two *cis* regulatory elements of CD44 gene: proximal promoter and the 3′UTR. We found that the effect was mediated by the 3′UTR, therefore we speculated that AR-42 may upregulate the expression of miRNA(s) targeting the CD44 3′UTR. Although our NanoString analyses identified many miRNAs that were differentially expressed in MM cells treated with AR-42, none of them was predicted to target the CD44 3′UTR. However, we discovered that the most consistently up-regulated miRNA, *miR-9-5p* contributed to CD44 expression indirectly, by targeting IGF2BP3, a protein described before to stabilize CD44 mRNA by binding to multiple sites in the CD44 3′UTR [31]. In addition, we cannot exclude that cryptic binding sites for miRNAs, or other regulatory RNAs can also participate in CD44 posttranscriptional regulation in MM cells. Previously published data have shown that the members of miR-*30* family decrease CD44 expression in MM cells at the transcriptional level through the downregulation of WNT/β-catenin pathway [57]. Interestingly, our miRNA profile and qRT-PCR validation data (data not shown) showed that miR-*30a* is significantly upregulated upon AR-42 treatment in MM cells. However, we did not observe transcriptional repression of the CD44 promoter in AR-42 treated MM cells, supporting the idea that CD44 downregulation upon AR-42 treatment could be driven by different mechanisms.

We also observed that miR-*9-5p* up-regulation was not exclusively achieved by the AR-42 treatment, but to a lesser extent and in dose-independent mannerit could be also modulated *in vitro* by other HDACi's, including SAHA and LBH589 (data not shown). We can speculate that in MM cells this miRNA is under epigenetic control and its re-expression is a more common mechanisms associated with the use of pan-HDACi's. In fact, transcriptional suppression of *miR-9-5p* by HDAC5 has been previously reported [63].

Recently published data show that that miR-*9-5p* is also targeting Blimp-1 [64–66]. Based on these data we can suppose that its expression can be beneficial in patients treated with lenalidomide, but can be a limiting factor for proteasome inhibitor (PI) treated patients, in which Blimp-1 downregulation has been associated with mechanisms of PI resistance [67].

CD44 expression is an important prognostic marker in MM, as well as other cancers and cancer stem cells [68]. Therefore, the use of AR-42 may allow CD44 targeting in numerous cancers that may both overcome resistance to standard therapeutic agents, as well as open up new treatment directions focused on cellular adhesion.

## MATERIALS AND METHODS

### Cell lines

MM cell lines MM.1S Include MM.1R and JJN3 myeloma cell lines, NCI-H929, KMS11, KMS18, OPM2, EJM, LP1, RPMI8226, U266 andL363 (courtesy of Dr. M. Kuehl; National Cancer Institute) were cultured in RPMI-1640 mediumsupplemented with 10% heat-inactivated fetal bovine serum (FBS). HeLa (CCL-2) and 293T (CRL-3216) were obtained from American Type Cell Collection (ATCC) and maintained in DMEM with 10% FBS.

### MM cell line transfections

One million of MM.1S, U266, or L363 cells were transfected by electroporation using Nucleofector 4D system (Lonza). Specific nucleofection slolutions and programs were optimized for each cell line. Briefly, cells were resuspended in 100 μl of the nucleofector solution SF, 30 pmols of microRNA (miR-*9-5p* precursor), antagomiR-9, miR-*16-5p*, negative control miR precursor, or siRNAs (Drosha, or scramble control) were added and transferred to a cuvette. All RNA reagents were from Life Technologies. Program DS-137 was used for MM.1S cells, program DN-100 for U266 and program DS-100 for L363. After electroporation, cells were immediately plated out in pre-warmed medium onto 6 well plates. AR-42 treatments were performed 24 hrs later.

### CD138+ plasma cell purification

CD138+ plasma cells (PCs) were purified from total bone marrow of patients by Human WholeBlood CD138+ Selection Kit (Cat#18387, Stem Cell Technologies), according to the manufacturer's instructions. Yield and purity of CD138+ cells was evaluated by flow cytometry using anti-CD138 antibody (Becton Dickinson).

### miRNA and mRNA profiling

Total RNA was prepared using TRIzol (Invitrogen). The RNA was analyzed by nCounter GX Human Immunology Kit, or nCounter Human microRNA Kit, as recommended by the manufacturer (NanoString Technologies, Inc.). A total of 511 immunology related genes and 800 microRNAs were profiled.

### Bioinformatic analyses

Samples analyzed by NanoString assay were normalized using the variance stabilization. The mean linkage hierarchical clustering algorithm was conducted to identify subgroups of significant miRNAs [69]. These results have been obtained using both the Rank Product package (version 2.16.0) of the BioConductor Library, under the R System and the Rank Product library in connection to the cluster analysis module of the Tmev system [70]. The obtained data were deposited in the GEO database (accession number).

### Enzyme-linked immunosorbent assay (ELISA)

Blood from xenografted mice (0.6 ml/kg) was collected by retro-orbital bleeding and serum was obtained by centrifuging it at 1500 × g for 10 min. ELISA was conducted as described by the manufacturer (Abcam). Briefly, serum was diluted 1:40 in Standard Diluent Buffer and 100 μl of each sample was plated in duplicate onto a 96-well plate. Standard and 1x control solution were added to the appropriate wells and incubated for 1 hr. All incubations were conducted at room temperature, unless otherwise noted. The plate was washed, biotinylated anti-CD44 added to each well and plate was incubated for 30 min. The plate was washed again and 100 μl 1x Streptavidin-HRP solution was added into each well, allowed to stand for 30 min. and washed again. Chromogen TMB substrate (100 μl) was added to each well and incubated in the dark for 15 min. Finally, 100 μl/well of Stop Reagent was added and absorbance was read on a spectrophotometer at 450 nm. Soluble CD44 (sCD44) content was calculated based on the readings from the standard and sample dilution factor.

### Immunoblotting

Cells were harvested by centrifugation, washed with PBS and lysed using buffer composed of 50 mM Tris (pH 7.5), 150 mM NaCl, 10% glycerol, 1.0% NP-40, 0.1% SDS, supplemented with protease and phosphatase inhibitors. Protein concentrations were estimated by Bradford assay and equivalent quantities of the lysates were resolved on 4–20% Tris-HCl SDS-PAGE TGX gels (Bio-Rad). Proteins were transferred to nitrocellulose membranes and stained for acetyl-histone H3 (Milipore), acetyl-histone H4 (Milipore), IGF2BP1 (Cell Signaling Technology), IGF2BP3 (IMP-3, Santa Cruz Biotechnology), CD44 (Santa Cruz Biotechnology), CD48 (Abcam), Drosha (Cell Signaling Technology), or glyceraldehyde 3-phosphate dehydrogenase (GAPDH, Cell Signaling Technology), followed by anti-mouse, or anti-rabbit IgG-HRP (GE Healthcare). Signals were developed using Pierce ECL Western Blotting Substrate (Thermo Fisher Scientific).

### DNA constructs

Human CD44 promoter-luciferase reporter gene (CD44P pGL3) [71] was obtained from Addgene (Plasmid 19122). The 3′UTR of CD44 was PCR amplified using following primers:

(Forward) 5′-gctagcCACCTACACCATTATCTTG-3′ and 5′-gctagcAATTCTTGGTGTTGTTATG-3′ (engineered NheI sites are in lower case), and the products were cloned into XbaI site downstream from the luciferase gene in pGL3-control vector (Promega). To generate IGF2BP3 luciferase reporter constructs, the 3′UTR was amplified by PCR using primers: (Forward) 5′-TCTTTGGTTATCTAGCTGTATGA-3′ and (Reverse) 5′-TCTTTGGTTATCTAGCTGTATGA-3′, and cloned into XbaI site of pGL3-control vector (Promega). Mutations in the miR-*9-5p* binding site of the IGF2BP3 3′UTR were introduced by the QuikChange Mutagenesis Kit (Stratagene) and the following primers:

(Forward) 5′-CAGAGGCAGATGCCAAACGGGG TACAGATTG CTTAACC-3′ and (Reverse) 5′-GGTTAA GCAATCTGTACCCCGTTTGGCATCTGCCTCTG-3′.

### Luciferase assay

Hela and 293T cells were transfected with 500 ng of 3′UTR-pGL3-control plasmid and 50 ng of Renilla luciferase expression construct (pRL-TK; Promega), using Lipofectamine 2000 (Invitrogen). After 24 hrs cells were lysed and tested by Dual Luciferase Assay (Promega), according to the manufacturer's instructions. MM.1S cells were transfected with 1.8 μg of pGL3-based luciferase vector and 200ng of pRL-TK, harvested 24 hrs later and assayed as above.

### mRNA and miRNA expression

Quantitative real time-PCR (qRT-PCR) was performed with the TaqMan method (Applied Biosystems), according to the manufacturer's instructions, and analyzed with the 7900HT Sequence Detection System (Applied Biosystems). The appropriate TaqMan probes for mRNA, miRNA, and pri-miRNA quantification were purchased from Applied Biosystems, and all reactions were performed in triplicate. The following probes were used: hsa-miR-*9-5p* (000583), hsa-mir-*9-1* (Hs03303201_pri), hsa-miR-*9-2* (Hs03303202_pri), hsa-mir-*9-3* (Hs03293595_pri), CD44 (Hs01075861_m1), IGF2BP3 (Hs00559907_g1). Simultaneous quantification of ornithine decarboxylase antizyme 1 (OAZ1), or GAPDH mRNAs were used as a reference for mRNA data normalization, while small endogenous nucleolar RNA U44/U48, or U6 were used for miRNA normalization. The relative expression levels were calculated by the comparative cycle threshold (Ct) method (User Bulletin #2; Applied Biosystems). Expression analyses of pre-miRNA was performed with SYBR green PCR master mix (Applied Biosystems) and normalized for U6 RNA. All primers used for amplification steps are listed in the Supplementary materials and methods.

### Flow cytometry

CD44 surface expression was analyzed by staining cells with CD44-FITC antibody (BD Biosciences) for 30 min. in the dark, at room temperature. Apoptosis was measured by Annexin V-FITC and Propidium Iodide (PI) (Clontech) staining for 15 min. in the dark, at room temperature and data immediately acquired on a Beckman Coulter FC500 (Beckman Coulter) machine. Analysis was conducted using the FlowJo Software vX.0.7 (Tree Star Inc.). For the multiparametric analysis, the bone marrow samples were stained with CD38-PE (347687; BD Bioscience), CD138-APC (347193; BD Bioscience), CD45-ECD (A07784; Beckman Coulter), CD44-FITC (BD Bioscience) and AnnexinV-FITC (Clontech) for 30 minutes, washed with PBS and immediately analyzed with Gallios cytometer (Beckman Coulter).

### Proliferation assay

Cell proliferation was assessed using the MTT cell proliferation assay (Promega) according to the manufacturer's protocol.

### Animal experiments

Animal experiments were performed according to OSU institutional guidelines. To generate MM xenograft model, 1 × 10^7^ viable MM.1S cells were injected subcutaneously into the right flank of 12 5-week-old female nude mice (Foxn1nu/Foxn1nu; Charles River). The tumor size was measured once a week using a caliper, and the volume was calculated in cubed millimeters (mm^3^), using the formula L × W^2^/2. At 3 weeks after injection, a group of 8 mice with comparable tumor size (250 ± 60 mm^3^) were randomly divided into two groups, using 4 mice for each treatment. Mice were treated with intra-tumoral injection of AR-42 (25 mg/kg) or DMSO (8% in PBS) once a day on Monday and Wednesday. The day after the second treatment, when the tumor sizes between the 2 different groups were comparable, blood from mice was collected by retro-orbital bleeding and the mice were sacrificed for IHC analysis.

For studies involving AR-42 combination with lenalidomide, GFP+/Luc+ MM.1S stable line [49] was harvested during logarithmic growth phase, washed with PBS and injected intravenously into NOD-SCID nude mice (5 × 10^6^ cells in 0.2 ml/mouse) under general anesthesia (isoflurane, 2–4% to effect). Beginning at 7 days post-injection, mice were monitored every day for the appearance of tumors by fluorescence using *in vivo* Imaging System (IVIS). On day 15, when the engraftment reached approximately ≥ 2 × 10^6^photons/sec/cm^2^/sr mice with similar tumor burden were divided into different groups of treatments. Intraperitoneal injections with vehicle control (8%DMSO in PBS), AR-42 (25 mg/kg; Mon-Tue-Fri) and lenalidomide (50 mg/kg, daily) were administered by intraperitoneal injection under general anesthesia (isoflurane, 2–4% to effect). Treatments for each mouse continued for 3 weeks, which ended when the control group showed sign of disease, including paralysis and extreme weight lost, or when tumor mass was equivalent to 10% of body weight.

### Detection of tumor progression by bioluminescence imaging

Mice were injected with 75 mg/kg Luciferin (Xenogen), and tumor growth was detected by bioluminescence 10 min. after the injection. The home-built bioluminescence system used an electron multiplying charge-coupled device (Andor Technology Limited) with an exposure time of 30 sec. and an electron multiplication gain of 500 voltage gain × 200, 5-by-5 binning, and with background subtraction. Images were analyzed using ImageJ software (National Institutes of Health).

### Immunohistochemistry

Xenograft tumor samples were fixed in 10% neutral-buffered formalin embedded in paraffin, and sectioned at 4 μm. Slides were then placed in a 60°C oven for 1 hr, cooled, deparaffinized, and rehydrated by passing slides through xylene, a series of graded ethanol solutions, and ending with water. All slides were placed for 5 min in a 3% hydrogen peroxide solution to block the endogenous peroxidase. Antigen retrieval was performed by heat induced epitope retrieval (HIER), in a citric acid solution, pH 6.1, for 25 min at 96°C followed by cooling down for 15 min. Slides were placed on a Dako Autostainer and sections were treated with primary antibodies for human CD138 and CD44 followed by biotinylated secondary antibodies and the DAB chromogen.

### Statistics

All preclinical data were obtained from at least three independent experiments and are expressed as mean ± standard deviation (SD). Comparisons between groups were performed using two-tailed *t*-tests, and comparisons between multiple groups were performed using 1-way analysis of variance (ANOVA).

Mouse data were evaluated by ANOVA, and synergy between AR-42 and Len was tested by interaction contrast. To investigate Annexin-V and CD44 level in primary patient samples, geometric mean values were analyzed by using mixed effect model and incorporated repeated measures for each sample. For the Annexin-V experiment, *p*-values were adjusted by Holm's method to control the familywise error rate at 0.05. Other *P* values reported in the manuscript were obtained by 2 tail *t*-test.

## SUPPLEMENTARY FIGURES AND TABLES


