# Correction: HDAC inhibitor AR-42 decreases CD44 expression and sensitizes myeloma cells to lenalidomide

**DOI:** 10.18632/oncotarget.28515

**Published:** 2023-09-25

**Authors:** Alessandro Canella, Hector Cordero Nieves, Douglas W. Sborov, Luciano Cascione, Hanna S. Radomska, Emily Smith, Andrew Stiff, Jessica Consiglio, Enrico Caserta, Lara Rizzotto, Nicola Zanesi, Volinia Stefano, Balveen Kaur, Xiaokui Mo, John C. Byrd, Yvonne A. Efebera, Craig C. Hofmeister, Flavia Pichiorri

**Affiliations:** ^1^Department of Internal Medicine, Comprehensive Cancer Center, The Ohio State University, Columbus, OH, USA; ^2^Department of Internal Medicine, Oncology/Hematology Fellowship, The Ohio State University, Columbus, OH, USA; ^3^Lymphoma and Genomics Research Program, IOR Institute of Oncology Research, Bellinzona, Switzerland; ^4^Department of Internal Medicine, Biomedical Sciences Graduate Program, Comprehensive Cancer Center, The Ohio State University, Columbus, OH, USA; ^5^Department of Internal Medicine, Biosystems Analysis, LTTA, Department of Morphology, Surgery and Experimental Medicine, Università degli Studi, Ferrara, Italy; ^6^Department of Neurological Surgery, Dardinger Laboratory for Neuro-oncology and Neurosciences, The Ohio State University Medical Center, Columbus, Ohio, USA; ^7^Department of Biomedical Informatics, Center for Biostatistics, The Ohio State University, Columbus, OH, USA; ^8^Department of Internal Medicine, Division of Hematology, The Ohio State University, Columbus, OH, USA; ^9^Present Address: Sanford Burnham Prebys Medical Discovery Insitute, La Jolla, CA, USA; ^*^These authors have contributed equally to this work

**This article has been corrected:** In Figure 3B, the legend and the text states that the myeloma cell line tested was MM.1S. However, the reported figure was for the derived drug resistant cell line MM.1R. The WB was run in both cell lines, but we reported MM.1R, since we observed CD44 regulation upon Drosha silencing only in the drug resistant cell line compared to the sensitive one. The Figure 3B legend has been corrected to MM.1R cells. The authors also clarify that the standard deviation of the densitometry in Figure 3B was calculated from high and low exposure times of one experiment showing different CD44 isoforms. The experiment was repeated *n* = 2 but only one is reported here. The corrected Figure 3, obtained using the original data, is shown below. The authors also replaced “MM.1S” with “MM1R” in the text on page 31137 of the paper. It should read, “Figure 3B demonstrates that inhibition of Drosha expression in MM.1R cells resulted in 2-fold increase of CD44 protein levels.” The authors declare that these corrections do not change the results or conclusions of this paper.

Original article: Oncotarget. 2015; 6:31134–31150. 31134-31150. https://doi.org/10.18632/oncotarget.5290

**Figure 3:**
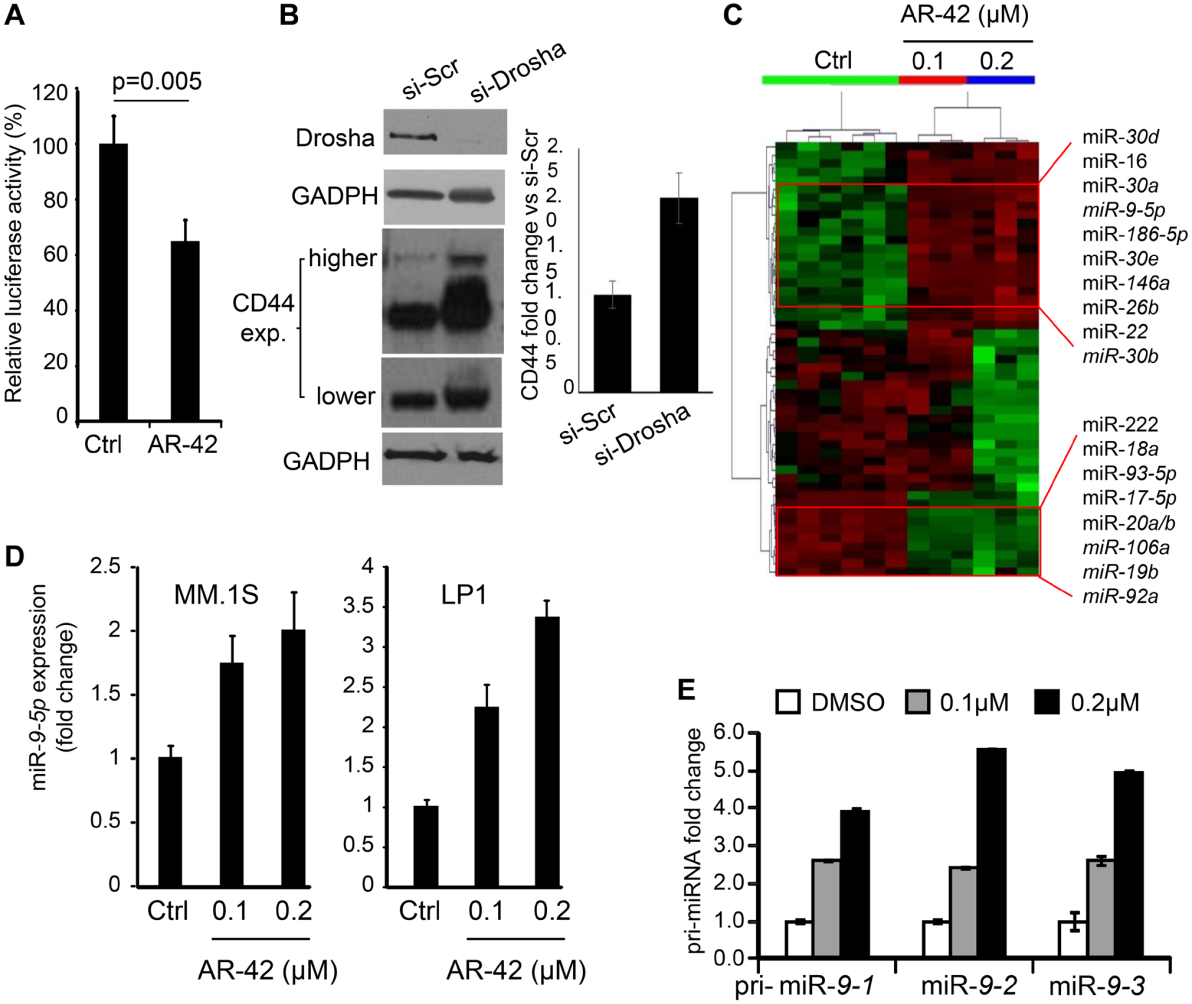
AR-42 upregulates expression of miR-9-5p. (**A**) Luciferase assay in MM.1S cells transiently transfected with pGL3-CD44 3′UTR construct and treated for 24 hrs with 0.2 µM AR-42, or vehicle control (Ctrl) showing inhibitory response to AR-42 via 3′UTR element. Each measurement was done in triplicate. (**B**) MM.1R cells were treated with RNA silencing for Drosha (si-Drosha) or unrelated sequence (si-Scr). Forty eight hours later, cells were lysed and analyzed by western blot using anti-Drosha and anti-CD44 antibodies. GAPDH was used for normalization of one experiment. Signals were quantified using ImageJ and plotted in the bar graph on the right. The standard deviation of the densitometry represents different CD44 isoforms visualized with higher and lower exposure (exp.) times and normalized for the GAPDH. (**C**) Dendrogram of the unsupervised hierarchical clustering analysis of global miRNA expression in MM.1S cells treated with designated concentrations of AR-42, or vehicle control (Ctrl), using NanoString technology. Selected most up-regulated (upper) and down-regulated (lower) miRNAs are indicated. (**D**) miR-9-5p expression in MM.1S (left) and LP1 (right) cells treated with AR-42 at 0.1 and 0.2 µM, or vehicle control (Ctrl) was determined by qRT-PCR. Results are expressed as fold change compared to the DMSO (Ctrl). (**E**) The effect of 24-hr treatment of MM.1S cells with AR-42 (at indicated concentrations) on expression of pri-miR-9-1, pri-miR-9-2 and pri-miR-9-3 was determined by qRT-PCR.

