# Supplementary material for: HDAC inhibitor AR-42 decreases CD44 expression and sensitizes myeloma cells to lenalidomide
Source: Oncotarget. 2015 Sep 25;6(31):31134–50. doi: 10.18632/oncotarget.5290 (PMC4741593; doi:10.18632/oncotarget.5290)
Supplement: Supplementary file 1 [file oncotarget-06-31134-s001.pdf]

SUPPLEMENTARY FIGURES AND TABLES

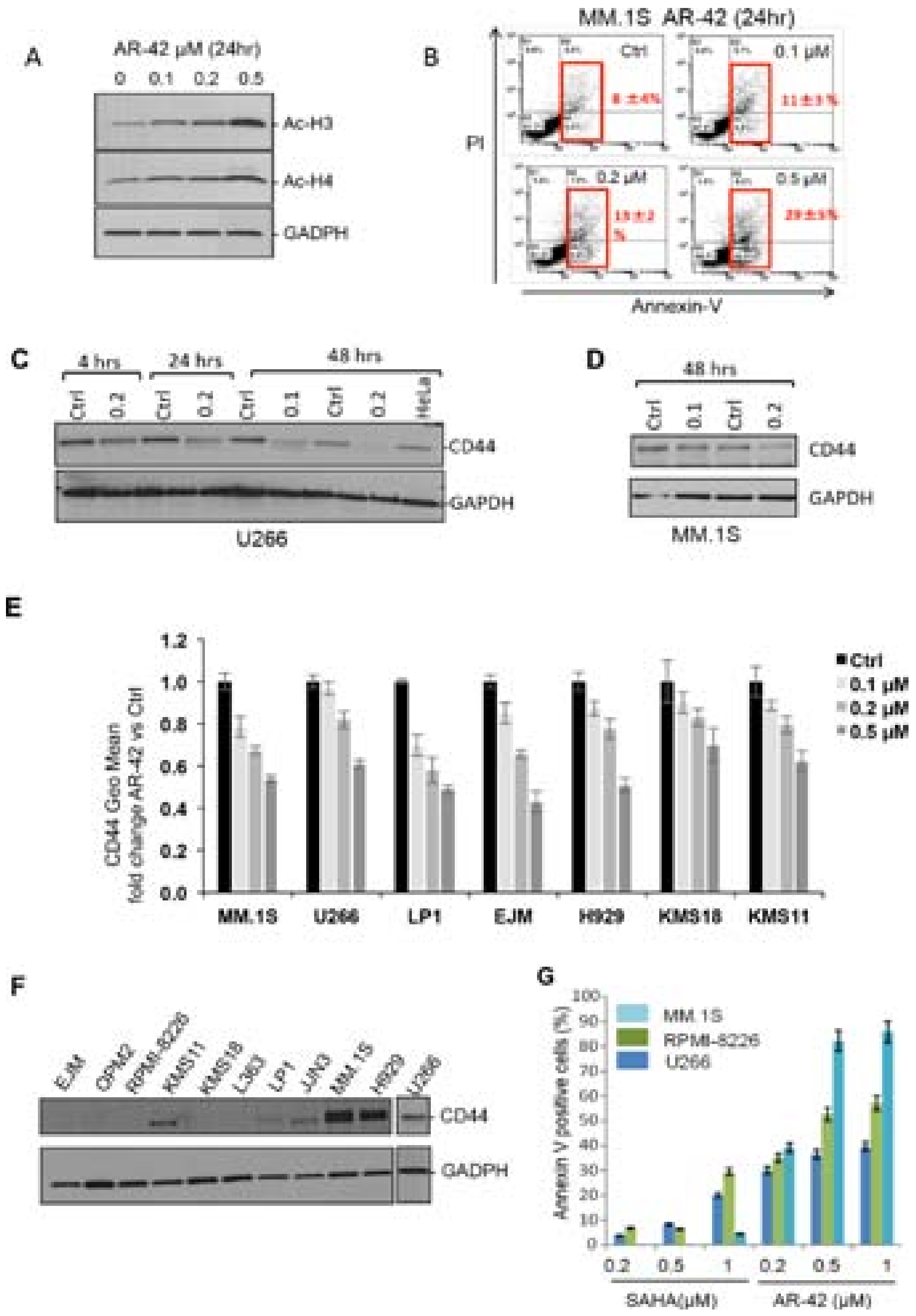

Supplementary Figure S1: *In vitro* effects of AR-42 in MM cell lines. A. Immunoblot analysis of acetyl histone H3 and H4 expression in MM.1S cells treated with 0.1–0.5 μM AR-42 for 24 hrs. GAPDH was used as a loading control. **B.** Induction of apoptosis in MM.1S treated with 0.1, 0.2 and 0.5 μM AR-42 for 24 hrs, measured by Annexin V-PI staining and flow cytometric analyses. The data are shown as mean ± SD of three independent experiments. **C–D.** CD44 expression by western blotting of U266 (C) and MM.1S (D) treated with indicated concentrations of AR-42 (μM). GAPDH was used as an internal loading control. HeLa cells were used as positive control. **E.** Flow cytometric analyses of CD44 expression in LP1, MM.1S, H929, EJM, KMS11 and U266 cell lines treated with AR-42 (0.1, 0.2 and 0.5 μM) for 48 hrs. Data are expressed as fold change of Annexin V geo mean compared to the untreated control. **F.** Western blotting analysis of the CD44 protein expression in EJM, OPM-2, RPMI-8226, KMS11, KMS18, L363, LP1, JJN3, MM.1S, H929 and U266 cell lines. GAPDH was used as loading control. **G.** Comparison of apoptosis induced by treatment of U266, RPMI-8226 and MM.1S cell lines treated for 48 hrs with SAHA (0.25, 0.5 and 1.0 μM), or AR-42 (0.25, 0.5 and 1.0 μM). Data expressed as percentages of Annexin V-FITC positive cells. (A-G) All data are representative of three independent sets of experiments.

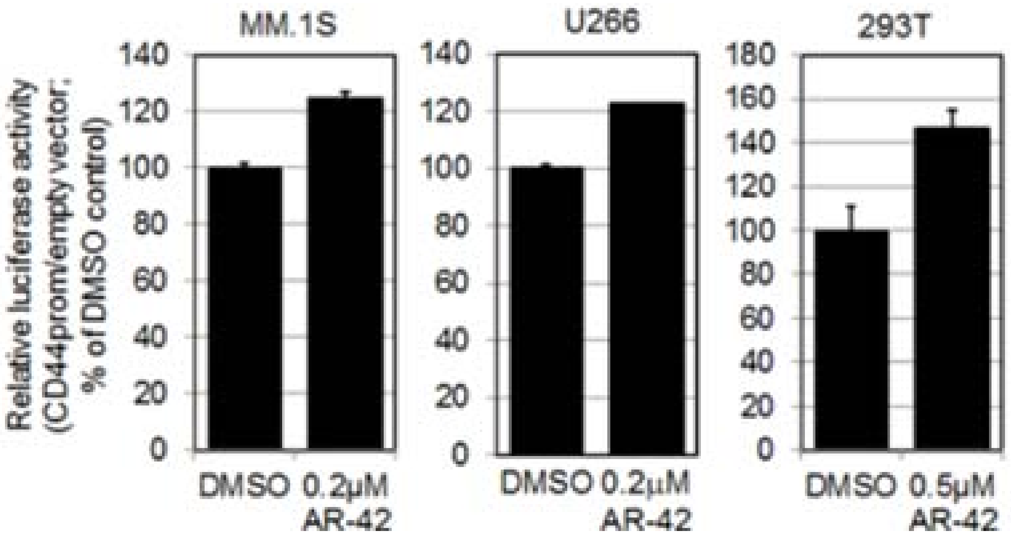

Supplementary Figure S2: Downregulation of CD44 by AR-42 is not mediated by the promoter region. MM.1S (left), U266 (middle) and 293T (right) cells were transfected with CD44P pGL3 and divided into two populations: one population was treated with vehicle control (DMSO), while the other with AR-42 at indicated concentrations. Cells were harvested 24 hrs later and luciferase activity was measured.

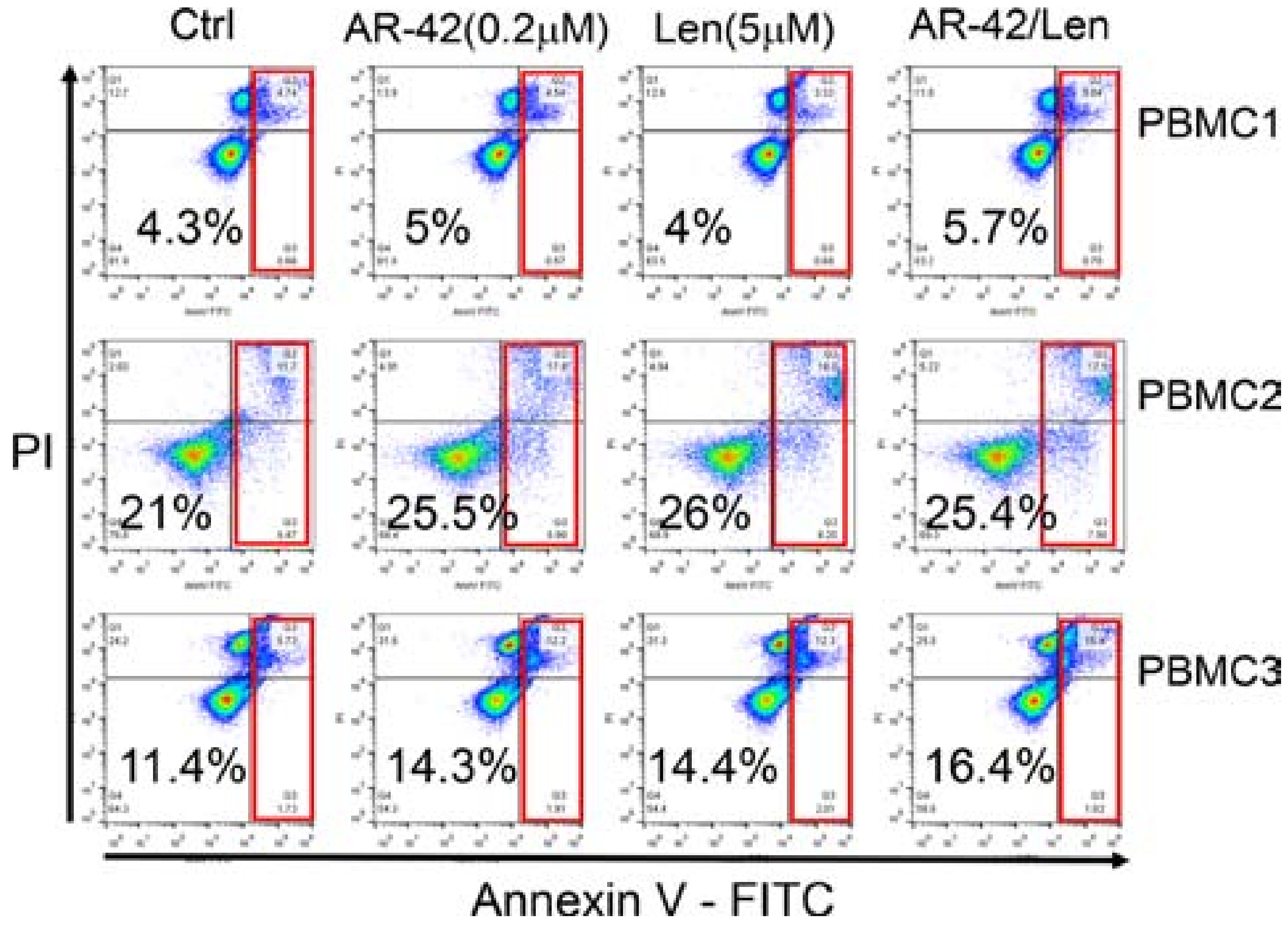

Supplementary Figure S3: AR-42 and Len alone, or in combination do not induce apoptosis of PBMCs. PBMCs from 3 MM patients were treated *ex vivo* with 0.2 µM AR-42 and 5 µM Len as single agents or in combination. After 48 hrs of treatment, cells were analyzed for apoptosis by Annexin V-PI staining using flow cytometer.

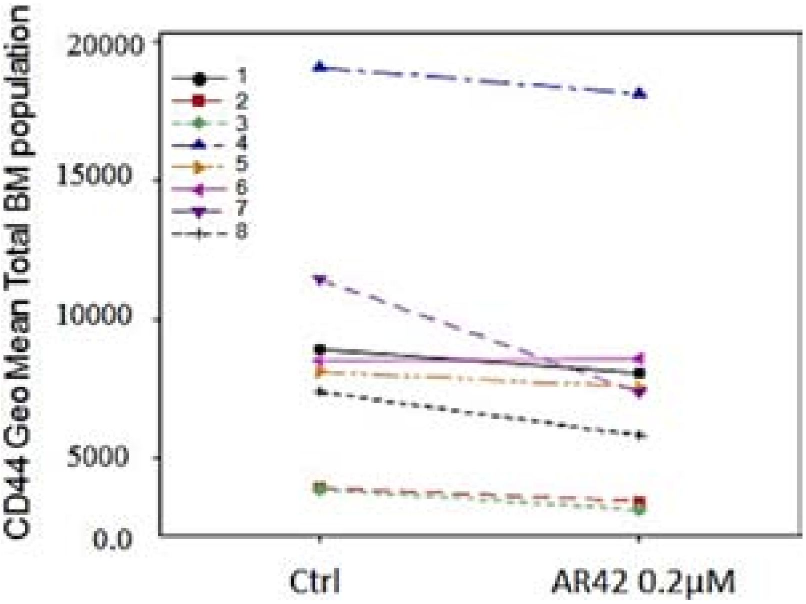

Supplementary Figure S4: Efficacy of AR-42/Len combination on CD44 levels in total BM. Total BM specimens from 5 Len-refractory and 3 newly diagnosed MM patients (same as in Figure 5C) were treated with 0.2 μM AR-42 for 48 hrs and evaluated for the expression of CD44 by flow cytometry. Values represent the CD44 geo mean.

Supplementary Table S1: Differentially expressed genes upon AR-42 treatment of MM.1S cells

Gene ID	log2_Fold Change AR-42 *vs.* Ctrl
IRF7	− 4.15
CD3EAP	− 3.60
ICOSLG	− 2.09
TFRC	− 1.12
MX1	− 1.04
CSF2RB	− 1.02
PTPN6	− 0.93
ADA	− 0.75
TUBB	− 0.73
TP53	− 0.63
SLAMF7	− 0.60
CD44	− 0.50
CD28	− 0.38
HLA-B	− 0.10
HLA-DRA	0.14
APP	0.17
CD164	0.33
CD99	0.41
IRF1	0.51
STAT3	0.53
TNFAIP3	0.60
LITAF	0.64
ITGB1	0.79
MAPKAPK2	0.85
IRAK4	0.89
CCL3	1.19
NFKBIZ	1.43
STAT4	1.95
PTPN22	2.20
CD40	2.32
CXCL10	2.49
CD81	5.44
ABCB1	5.57

Unsupervised hierarchical clustering analysis of nCounter® GX Human Immunology assays on MM.1S cells treated with AR-42 0.1 µM treatment for 24 hrs. Data are showed as expression of the majority of the immunology-related genes with ***p*** value < 0.001.

Supplementary Table S2: Differentially expressed miRNAs upon AR-42 treatment of MM.1S cells

microRNA_ID	0.1 µM *vs.* Ctrl	Adjusted *p*-values (0.1 µM *vs.* Ctrl)	0.2 µM *vs.* Ctrl	Adjusted *p*-values (0.2 µM *vs.* Ctrl)
hsa-miR-1973	11.45	1.13E-04	4.87	1.41E-04
hsa-miR-342-3p	9.74	1.75E-03	9.08	1.20E-04
hsa-miR-4516	8.09	1.94E-03	9.30	6.48E-05
hsa-miR-4284	7.29	2.20E-02	2.96	4.25E-02
hsa-miR-664-3p	6.06	6.53E-03	8.00	1.48E-04
hsa-miR-4485	5.92	2.18E-02	4.49	4.99E-03
hsa-miR-30a-5p	5.46	1.49E-02	4.65	1.91E-03
hsa-miR-575	5.28	1.51E-02	5.28	1.09E-03
hsa-miR-22-3p	5.22	9.03E-04	4.98	5.73E-05
hsa-miR-494	4.44	1.51E-02	5.22	5.08E-04
hsa-miR-630	4.19	1.64E-04	2.58	1.93E-04
hsa-miR-30d-5p	3.65	1.43E-03	2.83	3.98E-04
hsa-miR-146a-5p	2.70	1.13E-04	2.83	3.18E-06
hsa-miR-26b-5p	2.67	1.28E-05	2.73	2.42E-07
hsa-miR-320e	2.58	2.14E-02	2.14	6.35E-03
hsa-miR-361-3p	2.43	4.27E-03	2.67	1.31E-04
hsa-miR-30e-5p	2.27	1.30E-03	2.12	1.32E-04
hsa-miR-186-5p	1.95	1.30E-02	2.58	6.35E-05
hsa-miR-9-5p	1.82	4.93E-02	2.00	1.84E-03
hsa-miR-30b-5p	1.68	3.43E-03	1.43	2.84E-03
hsa-miR-548aa	0.46	1.09E-02	1.52	2.75E-02
hsa-miR-1244	0.45	3.45E-02	1.25	3.08E-01
hsa-miR-93-5p	0.44	1.12E-04	0.39	1.16E-06
hsa-miR-19b-3p	0.44	7.53E-05	0.49	6.25E-06
hsa-miR-20a-5p+20b-5p	0.41	1.13E-04	0.45	1.16E-05
hsa-miR-200c-3p	0.40	3.26E-03	0.91	5.73E-01
hsa-miR-365a-3p	0.39	3.26E-03	0.94	7.27E-01
hsa-miR-301a-3p	0.36	2.72E-04	0.49	2.04E-04
hsa-miR-18a-5p	0.36	7.75E-05	0.47	2.91E-05
hsa-miR-644a	0.35	2.58E-02	1.59	1.01E-01
hsa-miR-548ah-5p	0.33	3.06E-02	2.32	1.21E-02
hsa-miR-106a-5p+17-5p	0.33	5.10E-07	0.36	3.46E-08
hsa-miR-423-3p	0.33	1.51E-02	1.20	4.64E-01
hsa-miR-92a-3p	0.28	2.03E-04	0.33	2.77E-05
hsa-miR-4455	0.27	3.07E-02	1.18	6.35E-01

(Continued)

microRNA_ID	0.1 µM *vs.* Ctrl	Adjusted *p*-values (0.1 µM *vs.* Ctrl)	0.2 µM *vs.* Ctrl	Adjusted *p*-values (0.2 µM *vs.* Ctrl)
hsa-miR-223-3p	0.24	2.66E-02	1.22	5.83E-01
hsa-miR-335-5p	0.24	2.12E-02	1.57	2.05E-01
hsa-miR-4454	0.23	2.82E-02	0.78	5.24E-01
hsa-miR-720	0.21	3.45E-02	0.74	4.66E-01
hsa-miR-193b-3p	0.20	3.40E-04	1.43	1.07E-01
hsa-miR-450a-5p	0.18	3.26E-03	0.93	8.17E-01
hsa-miR-411-5p	0.17	5.84E-04	1.46	1.35E-01
hsa-miR-221-3p	0.13	2.76E-04	0.53	3.16E-02
hsa-miR-3676-3p	0.07	1.69E-05	0.60	3.89E-02
hsa-miR-126-3p	0.07	2.10E-07	0.30	3.77E-06

Unsupervised hierarchical clustering analysis of global miRNA expression in MM.1S cells treated by 0.1 µM, or 0.2 µM AR-42 for 24 hours and compared to DMSO treated cells (Ctrl).

Supplementary Table S3: Combinatorial index (CI) of AR-42 + Len

AR-42 (µM)	Len (µM)	CI
**0.1**	2.5	0.52
**0.1**	5.0	0.45
**0.1**	10.0	0.42
**0.2**	2.5	0.48
**0.2**	5.0	0.43
**0.2**	10.0	0.35

Proliferation MTT assay was performed on MM.1S cells treated with AR-42 (50 nM, or 100 nM) in combination with lenalidomide (Len; 1 µM, 2.5 µM, 5 µM, and 10 µM). Combinatorial indices (CI) were calculated by the Chou-Talalay method. All data are expressed as mean of three independent sets of experiments.
